# Duplicated middle cerebral artery associated with aneurysm at M1/M2 bifurcation: a case report

**DOI:** 10.1186/s13256-018-1824-7

**Published:** 2018-10-01

**Authors:** Kentaro Mori, Akira Tamase, Syunsuke Seki, Yu Iida, Yuichi Kawabata, Tatsu Nakano, Motohiro Nomura

**Affiliations:** 1 Department of Neurosurgery, Yokohama Sakae Kyosai Hospital, Yokohama, Japan; 2 Department of Neurosurgery, Kanto Rosai Hospital, 1-1 Kizukisumiyoshi-cho, Nakahara-ku, Kawasaki, 211-8510 Japan; 3 Department of Neurology, Yokohama Sakae Kyosai Hospital, Yokohama, Japan

**Keywords:** Anomaly, Duplicated middle cerebral artery, Aneurysm, Distal, M1/M2

## Abstract

**Background:**

A duplicated middle cerebral artery arises from the internal carotid artery and supplies blood to the middle cerebral artery territory. A duplicated middle cerebral artery is sometimes associated with an intracranial aneurysm. Most aneurysms associated with duplicated middle cerebral artery are located at the origin of the duplicated middle cerebral artery. An aneurysm located at the distal middle cerebral artery is not common.

**Case presentation:**

We encountered a 62-year-old Asian man with duplicated middle cerebral artery associated with aneurysms at the M1/M2 junction of the duplicated middle cerebral artery and top of the internal carotid artery.

**Conclusions:**

In cases of duplicated middle cerebral artery, association with a distal aneurysm on the duplicated middle cerebral artery is rare. However, the aneurysm may be formed on the thicker middle cerebral artery due to hemodynamic stress.

## Background

Some anomalies of the middle cerebral artery (MCA) have been reported on autopsy and radiological examinations [1]. Among them, a duplicated MCA (DMCA), accessory MCA, and fenestration are well known [1–3]. DMCA arises from the internal carotid artery (ICA) and supplies blood to the MCA territory. The incidence of DMCA has been reported to be 0.7–2.9% on autopsy and 0.24–1.5% on angiography [4].

There have been some reports describing cases of an anomalous MCA associated with an aneurysm [3, 5, 6]. Approximately 35 cases of DMCA associated with aneurysms have been reported in the literature [6–11]. Most of the cases had an aneurysm at the origin of the DMCA, and an aneurysm located at the distal DMCA was not common. To the best of our knowledge, only five cases of DMCA associated with distal MCA aneurysms have been reported [12–15]. Although the incidence is low, we should be aware of the association of DMCA and aneurysm.

We encountered a patient with DMCA and an aneurysm at the M1/M2 junction of the DMCA. This patient also had a small aneurysm at the top of the ipsilateral ICA. In this case report, we describe the patient with DMCA, and aneurysms on the distal DMCA and top of the ICA, and discuss the radiological findings and characteristics of the rare vascular anomaly and associated aneurysms.

## Case presentation

**Fig. 1:**
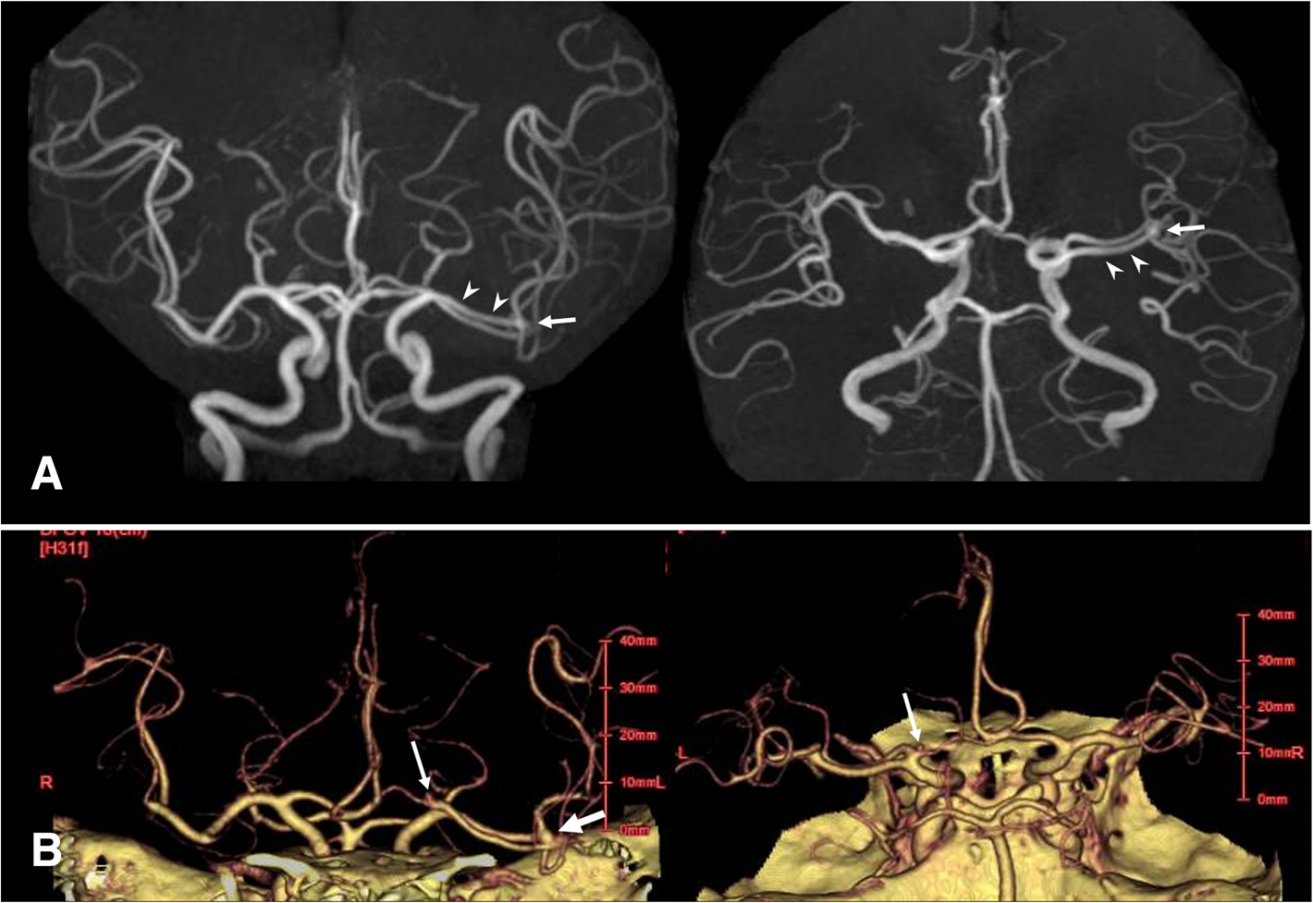
**a** Magnetic resonance angiography showing duplicated middle cerebral artery (*arrowheads*) on the left and an aneurysm at the M1/M2 junction of duplicated middle cerebral artery (*arrow*). **b** Three-dimensional computed tomographic angiography also demonstrating the left duplicated middle cerebral artery and aneurysms at the M1/M2 junction of duplicated middle cerebral artery (*arrow*) and internal carotid artery top (*small arrow*)

A 62-year-old Asian man with a medical history of diabetes mellitus and pancreatitis due to alcohol experienced speech disturbance. At the age of 58 years, he was treated with insulin for his diabetes mellitus. After that, his blood sugar level was well controlled by diet therapy. There were no relatives with intracranial aneurysms. His symptom was transient and had completely improved when he presented to our institution. He had no neurological abnormalities when he underwent radiological examinations. Magnetic resonance images showed no abnormality in his brain including hemorrhage or cerebral infarction. Magnetic resonance angiography (MRA) revealed a left DMCA that originated from the ICA distal to the anterior choroidal artery (Fig. 1a). An aneurysm at the M1/M2 junction of the DMCA was found (Fig. 1a). Three-dimensional computed tomographic angiography (CTA) also demonstrated the left DMCA associated with aneurysms at the M1/M2 junction and left ICA top (Fig. 1b). In our patient, the aneurysm was located on the DMCA, and not the main trunk of the MCA. The diameter of the DMCA was almost the same as that of the main MCA. The diameters of the DMCA and ICA top aneurysms were both less than 5 mm. Therefore, the aneurysms were not surgically treated, and periodic examinations by magnetic resonance images (MRI) and MRA were planned.

## Discussion

An aneurysm at the DMCA origin was initially reported in 1962 [8]. Most reported aneurysms related to DMCA were located at the DMCA origin [6, 7]. Aneurysms at locations other than the DMCA origin have also been reported [4, 6]. DMCA-related aneurysms have been suggested to be formed due to hemodynamic stress. To the best of our knowledge, 35 cases of DMCA origin aneurysm have been reported [6–11]. And almost half of the cases of DMCA aneurysms have multiple aneurysms due to hemodynamic condition [6]. Among them, only five cases of DMCA with distal MCA aneurysms have been reported [8–11]. These previously reported five cases and our case are summarized in Table 1. Among them, four aneurysms were located on the distal portion of the main MCA [12, 13, 15], and one aneurysm was located on the distal portion of the DMCA itself [14]. In our case, the aneurysm was found at the M1/M2 junction of DMCA, and not the main trunk of the MCA. The diameter of the DMCA was almost the same as that of the main MCA. Among the four reported cases with distal aneurysms on the main MCA, the main MCA trunk was thicker than that of the DMCA in three cases. In the other case, the diameters of the main MCA and DMCA were almost the same. On the other hand, in a case of an M1/M2 aneurysm on DMCA, the diameter of the main MCA was almost the same as that of the DMCA. These previous observations and our experience suggest that an aneurysm might be formed at the thick MCA due to hemodynamic factors. All the previously reported five cases were ruptured ones. The risk of rupture of distal DMCA aneurysm seems to be high. If the aneurysm is not small, radical treatment should be considered. For the three ruptured aneurysms, common treatment such as clipping was performed, and two patients showed good recovery. Furthermore, the distal ICA was tortuous in our case. The ICA top might be stressed by blood flow. Therefore, an aneurysm was formed at the top of the ICA due to hemodynamic stress. In the six cases of DMCA with distal MCA aneurysms, half of them had another aneurysm.

**Table 1:** Summary of cases of duplicated middle cerebral artery accompanied with distal aneurysms

Case	Age (years), sex	Ethnicity	Size of MCA (mm)		Location of aneurysm	Size of aneurysm (mm)	Rupture	Treatment	Outcome	Associated aneurysm or vessel anomaly	Reference
			MCA	DMCA							
1	67, F	Asian (Japanese)	0.71	0.37	M1 of main MCA	ND	+	ND	ND	–	[12]
2	66, M	Asian (Japanese)	0.92	0.32	M1/M2 of main MCA	ND	+	ND	ND	AComA aneurysm	[12]
3	55, F	ND	Both arteries were similar in size		Sylvian portion of the main MCA	ND	+	Clipping	Death	Contralateral MCA aneurysm (superior trunk)	[13]
4	45, F	Asian (Japanese)	Both arteries were similar in size		Distal trunk of DMCA	ND	+	Clipping	GR	Bilateral accessory MCA	[14]
5	34, F	Black	DMCA was smaller than the main MCA		Inferior division of main MCA	ND (> 10 mm in figure)	+	Clipping	GR	–	[15]
Present case	62, M	Asian (Japanese)	Almost the same		DMCA	3	_	Observation	No change	Ipsilateral ICA top aneurysm	

*AComA* anterior communicating artery, *DMCA* duplicated middle cerebral artery, *F* female, *GR* good recovery, *ICA* internal carotid artery, *M* male, *MCA* middle cerebral artery, *ND* not described

## Conclusions

In cases of DMCA, association with a distal aneurysm on DMCA is rare. However, the aneurysm may be formed on the thicker MCA due to hemodynamic stress.
